# Multidrug-Resistant *Enterobacter cloacae* Complex Emerging as a Global, Diversifying Threat

**DOI:** 10.3389/fmicb.2019.00044

**Published:** 2019-01-31

**Authors:** Medini K. Annavajhala, Angela Gomez-Simmonds, Anne-Catrin Uhlemann

**Affiliations:** Division of Infectious Diseases, Department of Medicine, Columbia University, New York, NY, United States

**Keywords:** carbapenem-resistant Enterobacteriaceae, carbapenem-resistant *Enterobacter cloacae* complex, carbapenemase, multidrug-resistance, bacterial genomics

## Abstract

The *Enterobacter cloacae* complex (ECC) includes common nosocomial pathogens capable of producing a wide variety of infections. Broad-spectrum antibiotic resistance, including the recent emergence of resistance to last-resort carbapenems, has led to increased interest in this group of organisms and carbapenem-resistant *E. cloacae* complex (CREC) in particular. Molecular typing methods based on heat-shock protein sequence, pulsed-field gel electrophoresis, comparative genomic hybridization, and, most recently, multilocus sequence typing have led to the identification of over 1069 ECC sequence types in 18 phylogenetic clusters across the globe. Whole-genome sequencing and comparative genomics, moreover, have facilitated global analyses of clonal composition of ECC and specifically of CREC. Epidemiological and genomic studies have revealed diverse multidrug-resistant ECC clones including several potential epidemic lineages. Together with intrinsic β-lactam resistance, members of the ECC exhibit a unique ability to acquire genes encoding resistance to multiple classes of antibiotics, including a variety of carbapenemase genes. In this review, we address recent advances in the molecular epidemiology of multidrug-resistant *E. cloacae* complex, focusing on the global expansion of CREC.

## Introduction

*Enterobacter spp.*, the second most common carbapenem-resistant Enterobacteriaceae (CRE) in the United States, increasingly contribute to the spread of carbapenem-resistant infections (Wilson et al., 2017). In particular, *Enterobacter cloacae* complex (ECC) are common nosocomial pathogens capable of producing a wide variety of infections, such as pneumonia, urinary tract infections, and septicemia (Sanders et al., 1997; Wisplinghoff et al., 2004). The emergence of multidrug resistance (MDR), including resistance to the last-resort carbapenems meropenem, imipenem, and ertapenem, has led to an increased interest in these organisms.

Molecular analyses based on multilocus sequence typing (MLST) and heat-shock protein (*hsp*) typing have led to the re-definition of members within this complex (Hoffmann and Roggenkamp, 2003; Paauw et al., 2008; Miyoshi-Akiyama et al., 2013). Whole-genome sequencing (WGS), moreover, has allowed for reproducible population-level analyses to determine clonal structure and diversity in ECC and CREC collections ranging from localized, regional outbreaks to global studies (Chavda et al., 2016; Gomez-Simmonds et al., 2018). These methods have facilitated analyses of phylogenetic structure and evolutionary history on a global scale.

Importantly, clinical and genomic studies have revealed a striking facility for ECC to acquire genes encoding broad-spectrum antibiotic resistance, including a variety of carbapenemase genes, superimposed on intrinsic β-lactam resistance conferred by chromosomal *ampC* genes. Here, we address recent advances in the molecular epidemiology, resistance mechanisms, global spread, and genomics of MDR ECC, focusing on CREC.

## Molecular Epidemiology of *E. cloacae* Complex

The *E. cloacae* complex is polyphyletic based on the traditionally employed 16S rRNA gene typing (Mezzatesta et al., 2012). Phenotypic methods and antibiotic susceptibility patterns were insufficient to resolve this genetically diverse species cluster. Molecular and genomic advances have enabled more refined species designations of ECC based on single amplicon (*hsp*60 or *rpoB*) genotyping, multilocus sequence analysis (MLSA), comparative genomic hybridization (CGH), pulsed-field gel electrophoresis (PFGE), and more recently, MLST and WGS. Based on *hsp*60 allelic variation, ECC was previously classified into thirteen genovars (clusters I-XIII). These encompass *Enterobacter asburiae* (cluster I), *Enterobacter kobei* (cluster II), *Enterobacter ludwigii* (cluster V), *Enterobacter hormaechei* subsp. *oharae* (cluster VI), subsp. *hormaechei* (cluster VII), and subsp. *steigerwaltii* (cluster VIII), *Enterobacter nimipressuralis* (cluster X), *E. cloacae* subsp. *cloacae* (cluster XI) and subsp. *dissolvens* (cluster XII), unnamed *E. cloacae* Hoffmann clusters III, IV, and IX, and an unstable *E. cloacae* sequence crowd (cluster XIII) (Brenner et al., 1986; Kosako et al., 1996; Hoffmann and Roggenkamp, 2003; Hoffmann et al., 2005a,b,c). However, using *hsp*60 or *rpoB* alone led to significant discrepancies in identification of subspecies (Paauw et al., 2008).

Multilocus sequence analysis based on 6 housekeeping genes (*rpoB, fusA, gyrB, leuS, pyrG*, and *rplB*) suggested the emergence of two distinct ECC clades: a recent clade including the three *E. hormaechei* subspecies and a heterogeneous older clade including multiple ECC clusters. The observed recombination:mutation ratio of 1.04 (95% confidence interval 0.72–1.45) across ancestral clades also indicates potential recombination events in the early evolution of ECC, likely accounting for discrepancies between single amplicon methods (Paauw et al., 2008). Based on MLSA, *Enterobacter mori* (Zhu et al., 2011), *Enterobacter xiangfangensis* (cluster VI), and *Enterobacter cancerogenus* were recently classified (Schonheyder et al., 1994). The remarkable genomic heterogeneity within ECC has even been used to suggest broad re-classification of the complex into five distinct genera based on MLSA (Brady et al., 2013). Despite ongoing debate regarding nomenclature within ECC, *E. cloacae* and *E. hormaechei* and related subspecies remain the most clinically relevant. In 2013, *dnaA* was added to the six genes of MLSA to develop an MLST scheme, which has emerged as a more robust tool for identifying closely related ECC isolates (Miyoshi-Akiyama et al., 2013). To date, 1069 sequence types (STs) have been reported.^1^

Comparison of the entire genome through WGS provides the opportunity to explore the genetic relationships between genomes at even higher resolution (Kluytmans-van den Bergh et al., 2016), and has further refined ECC classification into 18 clusters (A-R). These encompass the 12 Hoffmann clusters, *E. mori*, and five novel clusters (K, L, N, O, and P) (Chavda et al., 2016 and Supplementary Figure S1). Thus, the advent of WGS has greatly improved the ability to identify, investigate, and compare the emergence of ECC in diverse settings with high resolution, despite its polyphyletic and genomic diversity.

## Multidrug- and Carbapenem-Resistance in ECC

A variety of intrinsic and acquired antimicrobial resistance mechanisms have diminished the arsenal of effective therapeutics for treatment of ECC infections. ECC is intrinsically resistant to penicillins and first- and second-generation cephalosporins due to low-level expression of chromosomal *ampC* genes encoding an inducible AmpC-type Bush group 1 (class C) cephalosporinase. Resistance to third-generation cephalosporins and aztreonam can result from mutations, usually in *ampD*, leading to constitutive hyperproduction (derepression) of AmpC (Seeberg et al., 1983; Kaneko et al., 2005; Cheng et al., 2017).

Extended-spectrum β-lactamase (ESBL) genes confer resistance to most β-lactam antibiotics, including extended spectrum (i.e., second and third-generation) cephalosporins (ESCs) and monobactams (i.e., aztreonam). These genes are typically plasmid-encoded and were first identified in ECC in 1989 (De Champs et al., 1989). Since then, ESBL-encoding ECC have increased in prevalence, particularly in nosocomial settings and among patients with previous antibiotic exposure (Kluytmans-van den Bergh et al., 2016; Jean and Hsueh, 2017; Peirano et al., 2018). ESBL- and AmpC-mediated resistance now commonly coincide, leading to near-pan-resistance to β-lactams (Pitout et al., 1997).

Carbapenem-resistance in ECC is conferred through either constitutive overexpression of AmpC combined with disrupted membrane permeability, or more commonly through the acquisition of plasmid-encoded carbapenemase genes. Two major categories of carbapenemases have been identified in CREC, carbapenem-hydrolyzing serine β-lactamases (Ambler class A and D) and metallo-β-lactamases (MBLs; Ambler class B) (Supplementary Table S1). The *Klebsiella pneumoniae* carbapenemase (KPC), a class A β-lactamase which predominates in the United States, and the New Delhi metallo-β-lactamase-1 (NDM-1) have been most frequently described in ECC (Chavda et al., 2016), although substantial regional variation has been reported (Peirano et al., 2018). Rarely, ECC may also harbor chromosomally encoded carbapenemase genes (Boyd et al., 2017).

In addition to β-lactam resistance, ECC harbor a variety of multi-class antibiotic resistance genes. This includes aminoglycoside resistance primarily due to the acquisition of plasmids or mobile genetic cassettes encoding aminoglycoside 6’-N-acetyltransferase type I [AAC(6’)-I] (Neonakis et al., 2003). Mutations in DNA gyrase, DNA topoisomerase, or efflux pump genes have been associated with resistance to fluoroquinolones (Ruiz, 2003; Baucheron et al., 2004). Notably, ESBL and carbapenemase genes are often collocated with aminoglycoside-resistance genes on plasmids, engendering multi-class antibiotic resistance phenotypes (Chen et al., 2014; Chavda et al., 2016; Gomez-Simmonds et al., 2018).

An AAC(6′)-I variant produced by *aac*(6′*)-Ib-cr*, or the presence of plasmid-borne *qnr* or *qep* genes, can confer low-level quinolone resistance in ECC (Park et al., 2007; Périchon et al., 2007; Xiong et al., 2008; Cano et al., 2009; Kim et al., 2009). In addition, specific substitutions in chromosomal fluoroquinolone resistance-determining regions (QRDRs), such as the previously characterized double-serine/threonine substitutions in *gyrA* and *parC* (Hiramatsu et al., 2012), have been associated with improved fitness in major STs of other Enterobacteriaceae, including ESBL-producing *Escherichia coli* (Johnson et al., 2015) and *K. pneumoniae* (Tóth et al., 2014). This fitness advantage has been hypothesized to contribute to the spread of high-risk international STs while selecting against minor STs (Fuzi et al., 2017). QRDR mutations have been detected in ECC and appear to be widespread in CREC (Cano et al., 2009; Gomez-Simmonds et al., 2018; Guillard et al., 2015). However, their contribution to the spread of specific ECC and CREC clones has yet to be determined.

## Global Emergence of CREC

*E. cloacae* complex was one of the first KPC-producing organisms identified (Bratu et al., 2005), and has recently demonstrated an increase in prevalence and regional distribution (Park et al., 2016; Wilson et al., 2017). Current literature indicates that the emergence and spread of CREC is due to high diversity of clonal lineages and carbapenemases. A recent study leveraging two global surveillance programs demonstrated the remarkable dissemination and variety of carbapenemase genes in ECC (Peirano et al., 2018).

**FIGURE 1:**
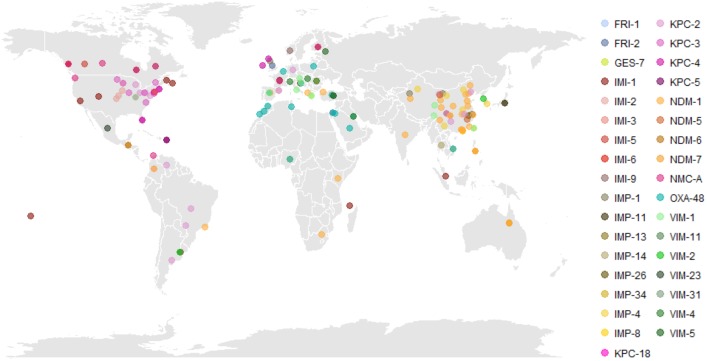
Global distribution of carbapenem-resistant *Enterobacter cloacae* complex (CREC). Literature review identified 61 English-language publications identifying carbapenemase subtypes in CREC with a specified geographic location of isolation. The regional emergence of carbapenemases is evident, with KPC and IMI predominant in North America, OXA-48 and VIM predominant in Europe and the Middle East, and NDM and IMP predominant in China and Southeast Asia. Underlying data and referenced publications can be found in Supplementary Table S2. Abbreviations: FRI, French imipenemase; GES, Guiana extended-spectrum β-lactamase; IMI, imipenem-hydrolyzing carbapenemase; IMP, active-on-imipenem carbapenemase; KPC, *K. pneumoniae* carbapenemase; NDM, New Delhi metallo-β-lactamase; NMC, non-metallo carbapenemase; OXA, oxacillinase; VIM, Verona integron-encoded metallo-β-lactamase.

We found 61 publicly available English-language publications identifying carbapenemase alleles in ECC with a corresponding geographic location (Supplementary Table S2). These encompassed 36 carbapenemase alleles (IMP-1,4,8,11,13,14,26,34; IMI-1,2,3,4,5,6,7,9; KPC-2,3,4,5,18; NDM-1,5,6,7; NMC-A; OXA-48; VIM-1,2,4,5,11,23,31; FRI-1,2; GES-7) in ECC from 44 countries, including single isolates and single or multi-institutional outbreak collections (Figure 1 and Supplementary Table S2). In the United States and Canada, *bla*_KPC_-positive ECC have been mostly encountered, with rare reports of IMI- and NMC-A-encoding organisms. Isolates harboring *bla*_KPC_ have also been detected in Europe and South America. While *bla*_NDM-1_ is endemic in the Indian subcontinent, multiple *bla*_NDM_ alleles were detected in hospitals throughout Eastern China (Jin et al., 2018; Wang et al., 2018). IMP-encoding genes have been reported widely in Southeast Asia, including China, Japan, Korea, the Philippines, Taiwan, and Australia, and are thought to be endemic to this area. On the other hand, VIM variants are more prevalent across Europe with rare reports from South America and Southeast Asia. OXA-48-like carbapenemases, thought to originate in Turkey, have spread into the Middle East, North Africa, and Europe (Poirel et al., 2011).

Previous multinational surveillance studies employing MLST found substantial clonal diversity of both ESBL-producing ECC and CREC, with evidence for several potential high-risk clones. The most widespread ESBL-producing ECC were ST66, ST78, ST108, and ST114, each having at least 10 isolates from three to five countries (Izdebski et al., 2015). Several epidemic clonal complexes (CC), such as CC74 (including ST78) or CC114 (including ST66) were identified, including specific ST66, ST78, and ST114 pulsotypes associated with carriage of CTX-M-15 β-lactamase. Likewise, ST114, (*E. xiangfangensis*), ST93 and ST90 (*E. hormaechei* subsp. *steigerwaltii*), and ST78 (*E. cloacae* cluster III) were widespread among global CREC isolates from 37 countries (Peirano et al., 2018), while ST105 (*E. xiangfangensis*) and ST108 were also identified in multiple countries.

## Genomic Insights Into the Spread of CREC Within the United States

While carbapenem-resistant *K. pneumoniae* (CRKP) appears to be declining in high-prevalence areas such as the Northeastern United States, multiple sites across the United States have reported increasing prevalence of CREC (Frieden et al., 2018). By 2015, over 4% of ECC clinical isolates collected in the United States Veteran’s Health Administration (VHA) nationwide were carbapenem non-susceptible, with especially high rates along the West Coast and Southwestern United States (Wilson et al., 2017). Most recently, New York City, Boston, Western Pennsylvania, North Carolina, and Minnesota/North Dakota have reported significant increases in CREC infections (Ahn et al., 2014; Hargreaves et al., 2015; Pecora et al., 2015; Gomez-Simmonds et al., 2016; Kanamori et al., 2017).

Limited information is available regarding specific genomic features of ECC potentiating its transmission and recent epidemiological success. However, the few available genomic studies suggest that establishment of successful clones as well as acquisition of MDR phenotypes by diverse lineages may have been substantial contributors.

ST171 has been identified as a major CREC clone with epidemic potential in the United States (Hargreaves et al., 2015; Chavda et al., 2016; Gomez-Simmonds et al., 2018). We previously found phylogenomic evidence that all ST171 with publicly available sequences formed two major clades which diverged and spread in parallel from the Northeastern to the Mid-Atlantic and Midwestern United States (Gomez-Simmonds et al., 2018). Our analysis estimated that these clades diverged prior to 1962, roughly two decades before the widespread use of carbapenems and fluoroquinolones, suggesting antibiotic pressure as a key factor in the proliferation of ST171.

ST171 is primarily associated with *bla*_KPC-3_, although a handful of *bla*_KPC-2_- and *bla*_KPC-4_-containing isolates have been identified. In the Northeast, CREC ST171 primarily contained a *bla*_KPC-3_ gene located on IncFIA plasmids (e.g., p34978, pNR3024) (Gomez-Simmonds et al., 2018). These plasmids were nearly identical to pBK30683, a ∼70 kb IncFIA plasmid which was widespread among *bla*_KPC_-producing *K. pneumoniae* in New York and New Jersey hospitals (Chen et al., 2014). Interestingly, a different study reported ST171 isolates from Minnesota and North Dakota which contained *bla*_KPC-3_ on a truncated (∼120 kb) IncFIA plasmid pMNCRE44 (Hargreaves et al., 2015). The truncated pMNCRE44 shared key regions with other ST171 IncFIA plasmids, but lacked genes encoding conjugation machinery. A small cluster of ST171 isolates from Boston instead contained *bla*_KPC-4_ on an unrelated IncHI2 plasmid (Pecora et al., 2015). A duodenoscope-mediated outbreak of CREC in a Michigan hospital also found likely patient-to-patient transmission of *bla*_KPC_-positive ST171 (KPC allele unreported) (Hawken et al., 2018). However, the hospital collection included diverse clones in which carbapenem-resistance was driven primarily by chromosomal mutations rather than carbapenemase genes. ST171 was rare in global surveys of both primarily carbapenem-susceptible (Girlich et al., 2015; Izdebski et al., 2015) and carbapenemase-producing ECC (Peirano et al., 2018), harboring three different carbapenemase genes presumably on different plasmid backbones. This suggests that stable uptake of the IncFIA plasmid by ST171 largely enabled its successful proliferation throughout the Northeastern United States, while isolates lacking this plasmid remain uncommon.

In contrast, ST78 was identified as a high-risk clone among both ESBL-producing ECC and CREC. CREC ST78 has largely been isolated in the Northeastern United States, with multiple sporadic uptake events of *bla*_KPC_-containing plasmids (Gomez-Simmonds et al., 2018), and has not exhibited the same rapid clonal proliferation as ST171. ST78 has been associated with various KPC-types on IncN plasmids, even within the New York City area (Gomez-Simmonds et al., 2018). Global carbapenemase-producing ST78 isolates have also been associated with a variety of plasmid backbones, highlighting its unique ability to acquire MDR plasmids. Peirano et al. (2018) demonstrated 4 different carbapenemases (*bla*_V IM-1_, *bla*_IMP-4_, *bla*_IMP-8_, *bla*_OXA-48_) on multiple different genetic backbones in ST78, although the carbapenemase-harboring plasmid could not be determined using short-read sequencing. In Japan, ST78 isolates harbored *bla*_IMP-1_ on class 1 integrons encoded on multiple different plasmids including IncHI2, IncW, and IncFIB (Aoki et al., 2018).

Other CREC STs have been associated with diverse KPC subtypes on IncN, IncX7, IncL/M, IncA/C, pKpQIL, and pKPC_UVA01-like plasmids, and plasmids with unknown replicon types (Chavda et al., 2016). However, few molecular studies include complete plasmid analyses, particularly for non-*bla*_KPC_ carbapenemases. Notably, although region-specific associations between carbapenemase genes and specific genetic backbones have been reported, shuffling of these genetic structures among different ECC clones appears to occur commonly including in geographically diverse areas (Peirano et al., 2018).

**FIGURE 2:**
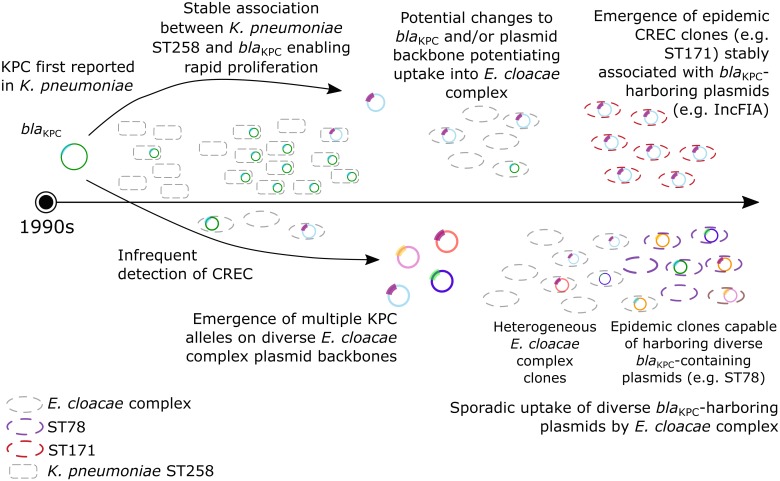
Emergence and spread of CREC. The first reports of KPC were in the mid-1990s. Carbapenem-resistant *K. pneumoniae* (CRKP) subsequently flourished due to a stable association between CRKP ST258 and *bla*_KPC_, although rare detection of CREC was reported. More recently, the apparent diversification of both KPC and plasmid backbones harboring *bla*_KPC_ may have enabled both (1) the emergence of epidemic CREC clones stably associated with *bla*_KPC_ -containing plasmids (i.e., ST171, red); (2) sporadic uptake of diverse *bla*_KPC_ -containing plasmids by heterogenous *E. cloacae* complex clones; and (3) emergence of epidemic clones capable of harboring diverse *bla*_KPC_-containing plasmids (i.e., ST78, purple).

Since the mid-1990s, when KPC was first described, the spread of CRKP has largely been attributed to the stable association between *bla*_KPC_ and the successful CRKP clone ST258 (Kitchel et al., 2009; Figure 2). Although isolated instances of CREC were reported around the same time, the diversification of both *bla*_KPC_ and plasmid backbones harboring these genes may have enabled uptake into diverse ECC. In contrast to CRKP, the spread of CREC can be attributed to not only stable *bla*_KPC_-clone associations, as in the case of ST171 and *bla*_KPC-3_-encoding IncFIA plasmids, but also the sporadic uptake of diverse plasmids by heterogeneous clones. This includes clones with epidemic potential capable of harboring diverse *bla*_KPC_-containing plasmid backbones, such as ST78.

## Other Genomic and Virulence Factors Potentiating the Spread of CREC

In addition to the presence of *bla*_KPC_ genes, other genomic factors linked to carbapenem- or other MDR may have aided in the rapid proliferation of CREC. Several lineage-specific genomic islands in both ST171 and ST78, encode for toxin-antitoxin and cell stress response systems (Gomez-Simmonds et al., 2018). Genes for toxin-antitoxin systems and heavy metal resistance have been found on MDR plasmids in CREC isolates (Aoki et al., 2018). These factors may further contribute to the success of this organism, particularly in nosocomial settings, although their specific impact on virulence and fitness has yet to be determined.

Virulence of CREC compared to carbapenem-susceptible ECC has not been extensively assessed. However, murine macrophage cytotoxicity assays did reveal significantly reduced cell killing of CREC vs. ESBL isolates and, more specifically, reduced toxicity of CREC ST171 vs. ESBL ST78 isolates (Gomez-Simmonds et al., 2018). Of note, no significant differences in cytotoxicity by site of collection or KPC-subtype were observed. Although previous studies reported detection of Shiga-like toxins in ECC (Paton et al., 1996; Probert et al., 2014), candidate genes were not detected in genomic analysis of these clones (Gomez-Simmonds et al., 2018). Thus, CREC may be a low-virulence pathogen with specific adaptations that enable success in nosocomial environments. In particular, cross-class antibiotic resistance and the acquisition of carbapenem- and fluoroquinolone-resistance determinants prior to the widespread use of these drugs point to the role of antibiotic pressure in hospital settings, rather than increased virulence, in the spread of CREC ST171 in the United States (Gomez-Simmonds et al., 2018). However, as previously suggested, potential fitness advantages conferred by QRDR mutations may play a role in the spread of major CREC STs, including ST171 and ST78, and should be evaluated further.

Analogous to the pan-genome, the concept of a “pan-metabolome” has also been applied to ECC (Rees et al., 2018). Several metabolite targets were identified, which discriminated between CREC and carbapenem-susceptible ECC, indicating a distinct metabolomic signature for each phenotype, beyond the presence of a single carbapenemase gene. The use of metabolomics and transcriptomics in future studies will be important to fully understand the complex relationships between genomic background, acquired carbapenemase resistance and virulence factors, and variable resistance phenotypes.

## Future Directions

Several gaps remain in our understanding of CREC. The notable diversity of CREC clones, carbapenemase genes, and plasmid backbones harboring MDR genes have thus far led to uncertainty regarding a clear timeline and evolutionary history of these organisms. Virulence, fitness, or other genomic factors potentiating the spread of CREC have not been completely defined or assessed *in vitro*. Moreover, despite recent advancements potentiated by WGS and comparative genomics, transcriptomics and/or metabolomics approaches may be useful in future studies to define the metabolic activity of CREC under different conditions. Lastly, the underreporting of CREC remains a possibility, and may influence findings regarding both population-level diversity and genomic mechanisms of resistance.

Regardless, the unique diversity of CREC, even compared to other CRE such as CRKP, necessitates a tailored approach to preventing its transmission and further diversification. The establishment of high-risk global CREC clones, coupled with the apparent high frequency of plasmid uptake into diverse ECC, suggests that vigilant tracking of both localized outbreaks and the potential for horizontal plasmid transfer is required.

## Author Contributions

A-CU initiated the review. AG-S performed a literature search. MKA wrote the first draft. All authors edited and reviewed the manuscript draft.

## Conflict of Interest Statement

A-CU has received research funding from Merck, GSK and Allergan, unrelated to the current study. The remaining authors declare that the research was conducted in the absence of any commercial or financial relationships that could be construed as a potential conflict of interest.

## Footnotes

1 http://pubmlst.org/ecloacae/

## Supplementary Material

FIGURE S1 Phylogenetic tree of representative *E. cloacae* complex (ECC) isolates showing relationships between Hoffmann clusters I-XII, genomic groups A-R, and selected sequence types (STs). At least one isolate with publicly available short-read sequences was selected from each ST previously reported in two recent genomic studies of CREC (Chavda et al., 2016; Gomez-Simmonds et al., 2018). NCBI Sequencing Read Archive (SRA) accession numbers are shown for each isolate in Supplementary Table S3. A public ST171 genome (GenBank CP012165) was used as the reference sequence for calling of concatenated core genome SNPs with snippy (https://github.com/tseemann/snippy) after removing mobile genetic elements and phage regions. The maximum likelihood tree was generated using RaxML with 100 bootstraps and visualized in iTOL (https://itol.embl.de/).

TABLE S1 Carbapenemase classes identified in carbapenem-resistant *Enterobacter cloacae* complex.

TABLE S2 Carbapenemase alleles by reported location.

TABLE S3 Metadata for selected isolates with publicly available whole-genome short-read data for phylogenetic analysis (Supplementary Figure S1).
