# Supplementary figures and images for: Multidrug-Resistant Enterobacter cloacae Complex Emerging as a Global, Diversifying Threat
Source: Front Microbiol. 2019 Jan 31;10:44. doi: 10.3389/fmicb.2019.00044 (PMC6365427; doi:10.3389/fmicb.2019.00044)

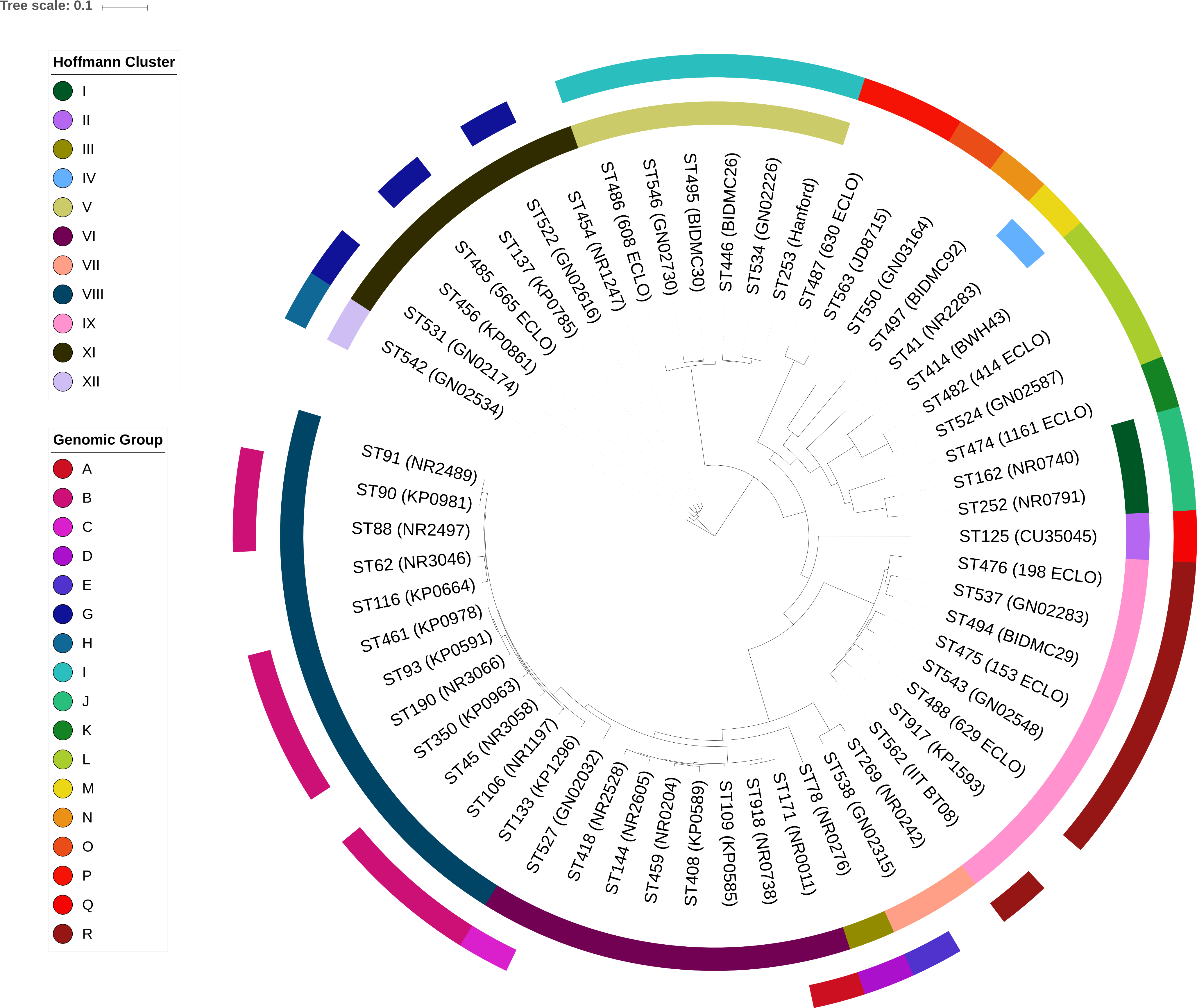

Supplement: FIGURE S1 — Phylogenetic tree of representative E. cloacae complex (ECC) isolates showing relationships between Hoffmann clusters I-XII, genomic groups A-R, and selected sequence types (STs). At least one isolate with publicly available short-read sequences was selected from each ST previously reported in two recent genomic studies of CREC (Chavda et al., 2016; Gomez-Simmonds et al., 2018). NCBI Sequencing Read Archive (SRA) accession numbers are shown for each isolate in Supplementary Table S3. A public ST171 genome (GenBank CP012165) was used as the reference sequence for calling of concatenated core genome SNPs with snippy (https://github.com/tseemann/snippy) after removing mobile genetic elements and phage regions. The maximum likelihood tree was generated using RaxML with 100 bootstraps and visualized in iTOL (https://itol.embl.de/). [file Image_1.TIF]
